# Microbe Profile: *Xylella fastidiosa* – a devastating agricultural pathogen with an endophytic lifestyle

**DOI:** 10.1099/mic.0.001091

**Published:** 2021-10-01

**Authors:** Lindsey P. Burbank, M. Caroline Roper

**Affiliations:** ^1^​ U.S. Department of Agriculture, Agricultural Research Service, San Joaquin Valley Agricultural Sciences Center, Parlier, California, USA; ^2^​ Department of Microbiology and Plant Pathology, University of California, Riverside, CA, USA

**Keywords:** biofilm, fastidious, insect transmission, xylem-limited

## Abstract

*
Xylella fastidiosa* is a vector-borne plant vascular pathogen that has caused devastating disease outbreaks in diverse agricultural crops worldwide. A major global quarantine pathogen, *X. fastidiosa* can infect hundreds of plant species and can be transmitted by many different xylem sap-feeding insects. Several decades of research have revealed a complex lifestyle dependent on adaptation to the xylem and insect environments and interactions with host plant tissues.

## Taxonomy

Phylum: Proteobacteria; class: Gammaproteobacteria; order: Xanthomonadales; family: *Xanthomonadaceae *; genus: *Xylella *; species: *X. fastidiosa *. Subspecies: fastidiosa*, multiplex*, pauca*, *sandyii*, *morus*. *Formally described in bacterial taxonomy.

## Properties

*X. fastidiosa* is a Gram-negative rod. The cells are typically 0.25–0.35 µm in diameter and 0.9–3.5 µm in length. The bacterium is non-flagellate, but motile via type IV pili-mediated twitching. *X. fastidiosa* lacks the type III secretion system typical of biotrophic plant pathogenic bacteria [1].

## Genome

The genome sequence of *X. fastidiosa* citrus-pathogenic strain 9a5c was published in 2000, making it the first genome sequence for a plant pathogenic bacterial species [2]. Complete genome assemblies for *X. fastidiosa* range from 2.5 to 2.7 Mb and many strains carry one–three plasmids of 1.2 to 51 kb. Expansion of available genome sequences to include a wide range of strains from different host plants has provided a tool for tracing new introductions worldwide. Currently there are publicly available genome assemblies for *X. fastidiosa* strains from multiple crop and ornamental hosts, and multiple countries.

## Phylogeny and genomic population structure

*X. fastidiosa* is divided into multiple subspecies groups, based on genetic differences. Subspecies *fastidiosa*, *pauca* and *multiplex* are formally described in bacterial taxonomy and represent genetic lineages originally associated with Pierce’s disease of grapevine, citrus variegated chlorosis, and various leaf scorch diseases, respectively [3]. The formally classified lineages are now also associated with additional diseases, such as almond leaf scorch (*fastidiosa*) and olive quick decline syndrome (OQDS, *pauca*) [4]. Other subspecies have been proposed for strains that infect oleander (subspecies *sandyi*) and mulberry (subspecies *morus*), but these have not been formally described in bacterial taxonomy [5]. In addition to disease events, strains of all subspecies can be found in asymptomatic hosts, or causing mild scorching symptoms that do not persist from 1 year to the next. To further define phylogenetic relationships of *X. fastidiosa* strains, a multi-locus sequence typing (MLST) system based on sequences of 7 housekeeping genes was widely adopted and 87 sequence types have been described to date [6]. More recently, whole-genome sequencing has emerged as the gold standard for strain classification.

## Key features and discoveries

Leaf scorching and die-back symptoms characteristic of *X. fastidiosa* infection are a combined result of bacterial growth within the vascular tissue and host defence responses that limit water flow in the plant. On initial infection, *X. fastidiosa* moves systemically through the xylem vessels facilitated by the secretion of cell wall-degrading enzymes and twitching motility using type IV pili. During the early stages of infection, plant response to *X. fastidiosa* invasion is delayed by the outer O-antigen portion of bacterial lipopolysaccharide surface structures that mask recognition by the innate immune system [7]. Outer-membrane vesicles (OMVs) are produced from the bacterial surface, which modulate adhesion to xylem cell walls and delay the formation of biofilms. As infection progresses, a diffusible signal factor (DSF) represses the production of OMVs once bacterial populations have reached higher densities, at which point *X. fastidiosa* attaches tightly to xylem vessel walls and forms biofilm structures dependent on the production of exopolysaccharide and fimbrial and afimbrial adhesins [1].

Horizontal gene transfer has been observed between strains and subspecies of *X. fastidiosa *, likely due to a high degree of natural competence for acquiring genetic material from the environment [5]. Natural transformation and homologous recombination occur at high frequency under *in vitro* conditions [8].

Despite the fact that specific *X. fastidiosa* strain–host plant combinations have resulted in devastating epidemics, in the majority of plant hosts *X. fastidiosa* does not cause severe disease. Specific factors that lead to parasitic rather than commensal interactions are not known, but some of the traits exhibited by *X. fastidiosa* during plant colonization contribute to virulence attenuation [7]. However, phenotypes necessary for insect transmission, such as the adhesive biofilm stage, also likely influence overall adaptation of *X. fastidiosa *.

Multiple insect species can transmit *X. fastidiosa* between plants through feeding on xylem sap. Insects of the leafhopper (Hemiptera: Cicadellidae) and spittlebug (Hemiptera: Aphrophoridae) families are the most common vectors associated with disease outbreaks [9, 10]. Insect–* X. fastidiosa* interactions lack specificity, with multiple insect vectors capable of transmitting *X. fastidiosa* to many hosts, and more than one strain of *X. fastidiosa* identified in the same insect [10]. Once acquired, *X. fastidiosa* adheres to the insect foregut, forming biofilms, and can persist for the life of the insect unless shed during moulting. Although *X. fastidiosa* multiplies within the insect vector, it does not enter the circulatory system and cannot be transmitted vertically to offspring [10]. As insect vectors of *X. fastidiosa* often have multiple feeding and reproductive host plants, the general nature of acquisition and transmission interactions contributes to the complexity of tracing and managing epidemics.

## Open questions

- What determines the host range of genetic lineages and specific strains of *X. fastidiosa *?
- Where does strain recombination occur (plant, insect)?
- How do secreted proteins and effectors influence *X. fastidiosa *–plant interactions?
- Do naturally occurring plasmids convey fitness benefits?
- To what extent does environmental adaptation of *X. fastidiosa* play a role in pathogen range and disease?

## References

[1] Rapicavoli J, Ingel B, Blanco-Ulate B, Cantu D, Roper C (2018). *Xylella fastidiosa*: an examination of a re-emerging plant pathogen. Mol Plant Pathol.

[2] Simpson AJG, Reinach FC, Arruda P, Abreu FA, Acencio M (2000). The genome sequence of the plant pathogen *Xylella fastidiosa*. Nature.

[3] Schaad NW, Postnikova E, Lacy G, Fatmi MB, Chang C-. J (2004). Xylella fastidiosa subspecies: X. fastidiosa subsp. [correction] fastidiosa [correction] subsp. nov., X. fastidiosa subsp. multiplex subsp. nov., and X. fastidiosa subsp. pauca subsp. nov. Syst Appl Microbiol.

[4] Saponari M, Boscia D, Altamura G, Loconsole G, Zicca S (2017). Isolation and pathogenicity of *Xylella fastidiosa* associated to the olive quick decline syndrome in southern Italy. Sci Rep.

[5] Nunney L, Schuenzel EL, Scally M, Bromley RE, Stouthamer R (2014). Large-scale intersubspecific recombination in the plant-pathogenic bacterium *Xylella fastidiosa* is associated with the host shift to mulberry. Appl Environ Microbiol.

[6] Yuan X, Morano L, Bromley R, Spring-Pearson S, Stouthamer R (2010). Multilocus sequence typing of *Xylella fastidiosa* causing Pierce’s disease and oleander leaf scorch in the United States. Phytopathology.

[7] Roper C, Castro C, Ingel B (2019). *Xylella fastidiosa*: bacterial parasitism with hallmarks of commensalism. Curr Opin Plant Biol.

[8] Kung SH, Almeida RPP (2011). Natural competence and recombination in the plant pathogen *Xylella fastidiosa*. Appl Environ Microbiol.

[9] Cornara D, Saponari M, Zeilinger AR, de Stradis A, Boscia D (2017). Spittlebugs as vectors of *Xylella fastidiosa* in olive orchards in Italy. J Pest Sci (2004).

[10] Krugner R, Sisterson MS, Backus EA, Burbank LP, Redak RA (2019). Sharpshooters: a review of what moves *Xylella fastidiosa*. Austral Entomology.

